# Genome Analysis Revives a Forgotten Hybrid Crop Edo-dokoro in the Genus *Dioscorea*

**DOI:** 10.1093/pcp/pcac109

**Published:** 2022-07-25

**Authors:** Satoshi Natsume, Yu Sugihara, Aoi Kudoh, Kaori Oikawa, Motoki Shimizu, Yuko Ishikawa, Masahiro Nishihara, Akira Abe, Hideki Innan, Ryohei Terauchi

**Affiliations:** Iwate Biotechnology Research Center, Kitakami, Iwate 024-0003, Japan; Crop Evolution Laboratory, Kyoto University, Mozume, Muko, Kyoto 617-0001, Japan; Crop Evolution Laboratory, Kyoto University, Mozume, Muko, Kyoto 617-0001, Japan; Iwate Biotechnology Research Center, Kitakami, Iwate 024-0003, Japan; Iwate Biotechnology Research Center, Kitakami, Iwate 024-0003, Japan; Crop Evolution Laboratory, Kyoto University, Mozume, Muko, Kyoto 617-0001, Japan; Iwate Biotechnology Research Center, Kitakami, Iwate 024-0003, Japan; Iwate Biotechnology Research Center, Kitakami, Iwate 024-0003, Japan; Laboratory of Population Genetics and Genome Evolution, The Graduate University for Advanced Studies, Hayama, Kanagawa 240-0193, Japan; Iwate Biotechnology Research Center, Kitakami, Iwate 024-0003, Japan; Crop Evolution Laboratory, Kyoto University, Mozume, Muko, Kyoto 617-0001, Japan

**Keywords:** Domestication, Genetic resource, Genome, Hybrid, Minor crop, Yam

## Abstract

A rhizomatous *Dioscorea* crop ‘Edo-dokoro’ was described in old records of Japan, but its botanical identity has not been characterized. We found that Edo-dokoro is still produced by four farmers in Tohoku-machi of the Aomori prefecture, Japan. The rhizomes of Edo-dokoro are a delicacy to the local people and are sold in the markets. Morphological characters of Edo-dokoro suggest its hybrid origin between the two species, *Dioscorea tokoro* and *Dioscorea tenuipes*. Genome analysis revealed that Edo-dokoro likely originated by hybridization of a male *D. tokoro* to a female *D. tenuipes*, followed by a backcross with a male plant of *D. tokoro*. Edo-dokoro is a typical minor crop possibly maintained for more than 300 years but now almost forgotten by the public. We hypothesize that there are many such uncharacterized genetic heritages passed over generations by small-scale farmers that await serious scientific investigation for future use and improvement by using modern genomics information.

## Introduction

The Food and Agricultural Organization (FAO) reported that only 30 plant species support 90% of the world calorie intake (FAO 1997). Three grain crops, rice, wheat and maize, account for more than half of global plant-derived energy intake. Accordingly, social and scientific attention has been focused on these major crops. The resulting dissemination of high-yield cultivars of rice and wheat to the world has enabled ‘Green Revolution’ in 1950–1960s by boosting higher production. However, it caused a loss of innumerable heterogenous traditional farmers’ varieties and other crops (Esquinas-Alcázar 2005), narrowing humans’ possibilities to sustain on divergent plant sources in various conditions. To prepare for future increases in food demand in ever-challenging global environments, preservation of extant genetic resources and their sensible use are crucial. Global efforts are needed to maintain the genetic diversity of crops and wild relatives worldwide ex situ and in situ (FAO 1997).

The so-called minor crops include staple crops for specific regions or localities. The typical minor crop includes yams representing tuber crops of the genus *Dioscorea*. More than 90% of world yam production comes from West and Central Africa that mainly grows Guinea yam (*Dioscorea rotundata*) (FAOSTAT 2018). In this region, Guinea yam is regarded as the ‘King of Crops’, important not only as food but as an integral socio-cultural component (Obidiegwu and Akpabio 2017, Obidiegwu et al. 2020). However, outside the region, it has not been given due scientific recognition. To contribute to enhancing yam production in the region, researchers have been studying genetic diversity and genomes of Guinea yam as reviewed by Sugihara et al. (2021). The first genome sequence of *D. rotundata* (Tamiru et al. 2017) set a stage for using genomics information for studies of *Dioscorea* and yams. The entire genus of *Dioscorea* is characterized by dioecy, with male and female flowers borne on separate individuals, which forces the taxa to complete outcrossing. Population genomics study of Guinea yam and its wild relatives revealed that *D. rotundata* is likely derived from a hybrid between the two wild species, *Dioscorea abyssinica* from the savannah and *Dioscorea praehensilis* from the rainforest, indicating the potential use of wild genetic resources for the improvement of Guinea yam (Sugihara et al. 2020). This example suggests that dioecy may have contributed to frequent hybridization in *Dioscorea* species as observed in *D. rotundata* (Terauchi et al. 1992, Scarcelli et al. 2006, Chaïr et al. 2010, Sugihara et al. 2020), *Dioscorea alata* (Chaïr et al. 2016) and *Dioscorea dumetorum* (Siadjeu et al. 2018).

The majority of yam crops belong to the section Enantiophyllum of *Dioscorea* (Arnau et al. 2010, Epping and Laibach 2020, Sugihara et al. 2021). However, there are many other *Dioscorea* species that may have contributed to human diet in the past, including *Dioscorea bulbifera* of the section Opsophyton (Martin 1974a, 1974b, Martin and Degras 1978a, 1978b). A large number of varieties exist in *D. bulbifera* worldwide that are seldom planted at the commercial scale, but several varieties are sporadically utilized as an emergency food (Martin 1974b, Terauchi et al. 1989, 1991). Another example is *Dioscorea tokoro* of the section Stenophora in Japan, which is commonly named ‘Tokoro’ or ‘Onidokoro’ in the region. The rhizomes of *D. tokoro* have been used as a food source or a famine food since long ago. A written record of trade of Tokoro dates back to AD 700s, on a wooden plate excavated from remains of ancient Nara town (Nara Ntl. Inst. For Cultural Properties 1990). In an agriculture book ‘Saifu’ in 1704 (Kaibara 1704), Ekiken Kaibara described that there is ‘Onidokoro’, which is acrid and nonedible, as well as ‘Tokoro’, which is edible and can be collected from the wild and cultivated in the field. He also described that ‘Tokoro’ from Edo, the former name of Tokyo, is yellow-colored and has a good taste. In a regional geography of the south part of Kyoto ‘Yoshu-Fushi’ in 1686 (Kurokawa 1686), Doyu Kurokawa described that there is a variety of ‘Tokoro’ called ‘Edo-dokoro’ that is big and sweet. By 1700, ‘Edo-dokoro’ has been locally cultivated as specialty, and a tasty variety of ‘Tokoro’ from Edo region was recognized, which may have been called ‘Edo-dokoro’. More recently, in a book of Japanese Flora, the botanist Tomitaro Makino (1940) described an edible variety of *D. tenuipes* with a name of ‘Edo-dokoro’. He described that ‘Edo-dokoro’ has been grown in Hachinohe city of Northern Japan. He personally obtained and grew its rhizome and reported that it was *D. tenuipes* (Satake et al. 1982). This note has been referred several times in other flora books, but there is no other recent information on the extant ‘Edo-dokoro’. Scientific investigations were needed to reveal its origin and to conserve the valuable genetic resources.

The recent development of genome analysis and population genomics has been providing an opportunity to examine the edible variety ‘Edo-dokoro’ in Japan. For *D. tokoro*, several studies showed genetic diversity (Terauchi 1990, Terauchi et al. 1997) and the genetic mechanism of sex determination (Terauchi and Kahl 1999). Recently, a chromosome-scale reference genome of *D. tokoro* (2*n* = 2*x* = 20) was obtained (Natsume et al. 2022). In this study, we show that four farmers in Aomori prefecture in Japan have been serving as guardians of this long-forgotten genetic heritage ‘Edo-dokoro’ and discuss the origin of ‘Edo-dokoro’ in relation to *D. tokoro*, based on morphological and genome analyses.

## Results

### Encounter with Edo-dokoro crop

**Fig. 1:**
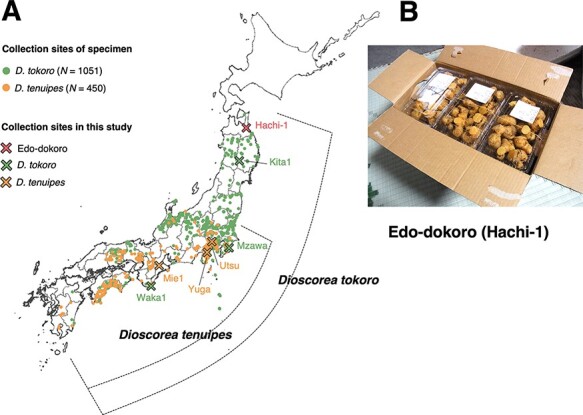
Geographical distributions of *D. tokoro* and *D. tenuipes* and the location of Edo-dokoro (Hachi-1) cultivation. (A) The map shows the collection sites of herbarium specimen of *D. tokoro* and D. tenuipes as well as Hachi-1 sample. *Dioscorea tokoro* is distributed widely in Japan, from Kyushu Island to North Honshu Island. On the other hand, D. tenuipes is not distributed in the northern part of Japan including the Aomori prefecture that cultivates Hachi-1 sample. The distribution limits and range showed by dotted line were adopted from Okagami and Kawai (1982). Green and yellow dots represent the sampling sites of the specimens of *D. tokoro* and *D. tenuipes*, respectively. Specimen data were provided from the S-Net data portal (https://science-net.kahaku.go.jp). Red cross (Hachi-1), green crosses (Kita1, Mzawa, Waka1) and yellow crosses (Utsu, Yuga, Mie1) represent the sampling sites of Edo-dokoro, *D. tokoro* and *D. tenuipes*, respectively. The map was created with Quantum Geographic Information System (QGIS) software version 3.16.0 (QGIS Development Team 2022). The base map was obtained from the Geospatial Information Authority of Japan, 2020. (B) The rhizomes of Edo-dokoro sold in Hachinohe market.

We noticed that the rhizomes of wild plants of *D. tokoro* are locally consumed in the Northern Honshu Island of Japan. To obtain the information on *D. tokoro* consumption in the region, we visited local markets in Aomori prefecture in 2014. In a market at Hachinohe city (**Fig. 1**; **Supplementary Fig. S1**), we found boiled rhizomes of a *Dioscorea* species with the name tag ‘Tokoro’. The rhizomes were highly branching with oblong branches and were sweet and not acrid in taste. These characters differed from typical *D. tokoro* having less branched rhizomes (Makino 2008) and acrid taste presumably because of saponins (Oyama et al. 2017). We succeeded in meeting farmers at Tohoku-machi town (**Supplementary Fig. S1**) producing the crop, and we obtained the rhizomes that have been clonally propagated. Morphology of aboveground and underground parts of the plant has typical characteristics of *D. tenuipes*. These characteristics and the producing region matched the reports of the edible variety ‘Edo-dokoro’ in Makino (1940) and Satake et al. (1982); hence, we call the crop Edo-dokoro hereafter.

Currently, only four families are producing and inheriting Edo-dokoro (**Supplementary Fig. S2**). They place small pieces of rhizomes into soil in spring and let them grow by training vines upward that are supported by metal stakes. In November, the rhizomes are harvested, and roots are removed. The ratio of growth from seed rhizomes to harvested rhizomes is about 12-fold. After boiling the harvested rhizomes for 2–4 h, they are cleaned and cut into small pieces and put into plastic containers. The products are brought to a local wholesale market for auction and sold to retailers. One of such retailers is a supermarket in Hachinohe. The boiled rhizomes are consumed as a kind of niche food by the local people.

### Botanical characteristics of Edo-dokoro suggest its link to *D. tokoro* and *D. tenuipes*

The rhizome of Edo-dokoro was brought back to the Iwate Biotechnology Research Center (IBRC), Kitakami, Iwate, and grown in its greenhouse. The botanical characteristics of Edo-dokoro were compared with those of *D. tokoro* and *D. tenuipes*, which were candidate ancestors of Edo-dokoro. These two candidate species have different geographical distributions (**Fig. 1**; Okagami and Kawai 1982). *Dioscorea tokoro* is widely distributed in Japan, whereas *D. tenuipes* is distributed only in the central and western parts of Japan where Edo-dokoro is not cultivated.

**Fig. 2:**
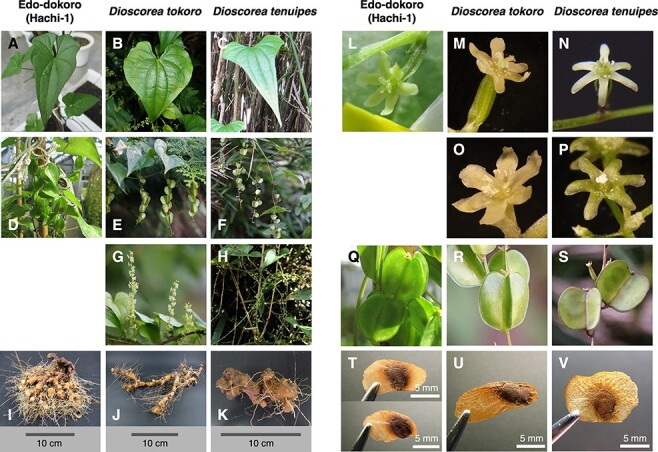
Botanical characteristics of Edo-dokoro (Hachi-1) suggest its link to both *D. tokoro* and *D. tenuipes*. (A) Leaf of Edo-dokoro (Hachi-1). (B) Leaf of *D. tokoro*. (C) Leaf of *D. tenuipes*. (D) Pendent female inflorescences of Edo-dokoro (Hachi-1) with mature capsular fruits. (E) Pendent female inflorescences of *D. tokoro* with mature capsular fruits. (F) Pendent female inflorescences of *D. tenuipes* with mature capsular fruits. (G) Upright male inflorescences of *D. tokoro*. (H) Pendent male inflorescences of *D. tenuipes*. (I) Rhizome of Edo-dokoro (Hachi-1) with thick and short branch. (J) The rhizome of *D. tokoro* with horizontal growth and long branch. (K) The rhizome of *D. tenuipes* with horizontal growth and short branch. (L) Female flower of Edo-dokoro (Hachi-1). (M) Female flower of *D. tokoro*. (N) Female flower of *D. tenuipes*. (O) Male flower of *D. tokoro*. (P) Male flower of *D. tenuipes*. (Q) Capsular fruits of Edo-dokoro (Hachi-1). These fruits were obtained after crossing with pollen from *D. tokoro*. (R) Capsular fruits of *D. tokoro*. (S) Capsular fruits of *D. tenuipes*. (T) Winged seeds in fruits of Edo-dokoro (Hachi-1). These seeds were produced from crosses with pollen from *D. tokoro*. Upper figure shows the seed, winged in both basal and apical positions, on the stigma side of capsular. Lower figure shows the seed, winged apical only, on the pedicel side. (U) Seed of *D. tokoro* winged apical only. (V) Seed of *D. tenuipes* winged all around.

We found that the botanical characteristics of Edo-dokoro are not clear enough to classify it as either *D. tokoro* or *D. tenuipes*. The leaf of Edo-dokoro is oblong, whereas it is oval in *D. tokoro* and oblong in *D. tenuipes* (**Fig. 2A**–**C**; Makino 2008). The rhizome of Edo-dokoro has a distinctive morphology from those of *D. tokoro* and *D. tenuipes*. The short branches of Edo-dokoro (**Fig. 2I**) are similar to those of *D. tenuipes* (**Fig. 2K**; Kiyosawa and Kawasaki 1975) rather than the long branches of *D. tokoro* (**Fig. 2J**; Makino 2008). Also, its spherical shape is similar to that of *D. tenuipes* rather than the stick shape of *D. tokoro*. However, each spherical and short branches is densely clumped, forming the rhizome of Edo-dokoro (**Fig. 2I**). After flowering, the Edo-dokoro plant turned out to be female. Morphology of female flowers of Edo-dokoro is more similar to that of *D. tokoro* with flat and wide petals than that of *D. tenuipes* with narrow petals (**Fig. 2L**–**N**; Akahori 1965; Ohwi and Kitagawa 1992; Makino 2008). To perform the quantitative comparison of the morphological characters of Edo-dokoro, *D. tokoro* and *D. tenuipes*, we measured the size of their capsular fruits and seeds using 70–80 individuals of the respective taxa (**Supplementary Table S1**). Length and width of capsules of Edo-dokoro are in between those of *D. tenuipes* and *D. tokoro* (**Fig. 2Q**–**S**, **Supplementary Fig. S3**; Ohwi and Kitagawa 1992). Seeds of Edo-dokoro, *D. tokoro* and *D. tenuipes* all have wings. Wing width and wing height of Edo-dokoro are in between those of *D. tokoro* and *D. tenuipes* (**Fig. 2T**–**V**, **Supplementary Fig. S4**). Wing width of the pedicel side is highly variable in Edo-dokoro (**Supplementary Fig. S4**), with the majority of seeds appearing similar to *D. tenuipes* but others to *D. tokoro* (**Fig. 2T**–**V**). As a result, our morphological assessment of Edo-dokoro indicates that this plant has the characteristics of both *D. tokoro* and *D. tenuipes*.

### Flow-cytometry analysis suggests that Edo-dokoro is diploid

**Fig. 3:**
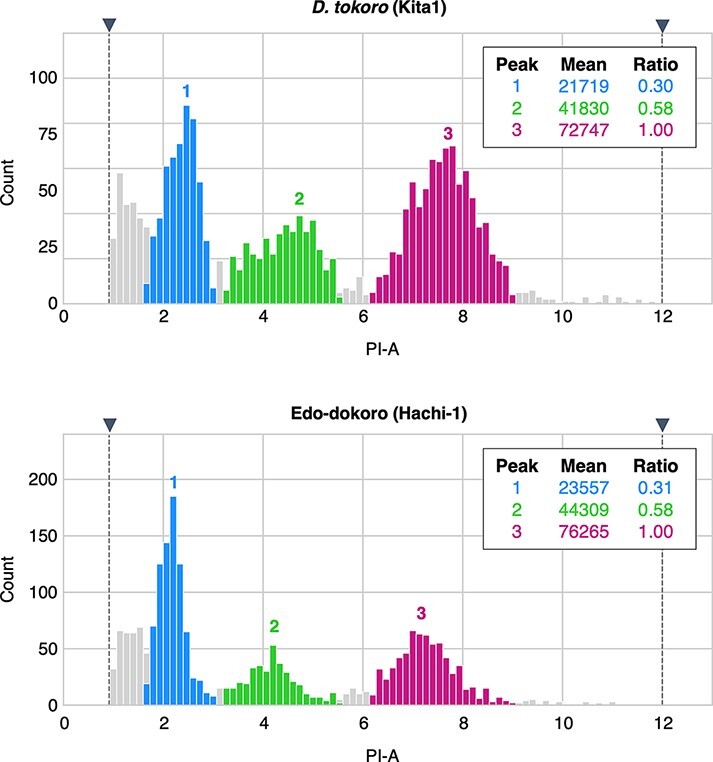
Flow-cytometry analysis shows that the ploidy of Edo-dokoro is the same as that of *D. tokoro*. Histogram of relative fluorescence intensity of nuclei shows three peaks: 1 = G0/G1 phase of *D. tokoro/*Edo-dokoro, 2 = G2 phase of *D. tokoro/*Edo-dokoro and 3 = *P. hybrida* cv. Mitchell. G1 and G2 indicate the gap 1 and gap 2 phases of the cell cycle.

At least 33% of the investigated *Dioscorea* species have variable ploidy levels within the same species (Sugihara et al. 2021). Therefore, before starting the genome analysis, we evaluated the DNA content of Edo-dokoro (Hachi-1) as compared with those of *D. tokoro* (2*n* = 2*x* = 20; 443 Mb, Natsume et al. 2022) and *Petunia hybrida* (~1.4 Gb; White and Rees 1987; Bombarely et al. 2016) by flow-cytometry analysis (**Fig. 3**). If the DNA content of *P. hybrida* was set to 1.00 (1,400 Mb), those of *D. tokoro* and Edo-dokoro were 0.30 (∼420 Mb) and 0.31 (∼434 Mb), respectively, showing that the genome size of Edo-dokoro is similar to that of *D. tokoro*. This suggests that Edo-dokoro is a diploid.

### Genome analyses of Edo-dokoro reveal its hybrid origin

We set out to elucidate the origin of Edo-dokoro using genome sequence information. For this purpose, we used a reference genome sequence of *D. tokoro* (Natsume et al. 2022). The total size of *D. tokoro* genome was 443 Mb distributed over 2,931 contigs (*N*_50_ = 586.3 kb). The contigs were anchored on linkage groups, resulting in 10 pseudochromosomes. We sequenced Edo-dokoro (Hachi-1) with the candidate ancestors *D. tokoro* and *D. tenuipes*. For *D. tokoro*, we used the whole-genome sequences of three individuals (Kita1 from Kitakami, Iwate; Waka1 from Kushimoto, Wakayama; and Mzawa from Mutsuzawa, Chiba; **Fig. 1**; **Supplementary Table S2**). For *D. tenuipes*, we used the whole-genome sequences of three individuals (Mie1 from Misugi, Mie; Yuga from Yugawara, Kanagawa; and Utsu from Hachioji, Tokyo; **Fig. 1**; **Supplementary Table S2**). As an outgroup, we used the whole-genome sequence of a distantly related species, *Dioscorea quinqueloba* (**Supplementary Table S2**). We compared the whole-genome sequence of Edo-dokoro with those of *D. tokoro* and *D. tenuipes*, using the *D. tokoro* reference genome.

First, we calculated the nucleotide diversities of *D. tokoro* and *D. tenuipes*, using 3,113,287 and 1,496,981 single nucleotide polymorphisms (SNPs), respectively (**Supplementary Table S3**). The nucleotide diversity of *D. tokoro* was 0.00392 and that of *D. tenuipes* was 0.00376. Then we calculated the *F*_ST_ between *D. tokoro* and *D. tenuipes*, which was 0.534. Considering that the *F*_ST_ between *D. abyssinica* and *D. praehensilis*, the wild ancestral species of the hybrid crop *D. rotundata* was 0.162 (Sugihara et al. 2020); the *F*_ST_ between *D. tokoro* and *D. tenuipes* indicates that the populations of *D. tokoro* and *D. tenuipes* are more diverged than those of *D. abyssinica* and *D. praehensilis*.

**Fig. 4:**
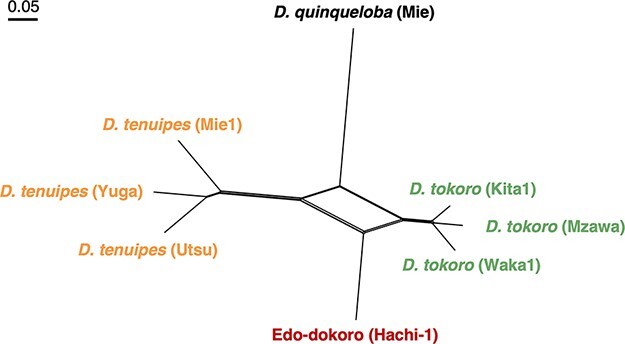
Edo-dokoro (Hachi-1) is not classified as either of *D. tokoro* or *D. tenuipes* based on the neighbor-net. The neighbor-net was reconstructed by SplitsTree5 (Huson and Bryant 2006). *Dioscorea quinqueloba* is an outgroup of this tree.

We reconstructed a neighbor-net (Huson and Bryant 2006) using a total of 1,054,866 SNPs that are biallelic among the eight samples. The result showed that three *D. tokoro* samples and three *D. tenuipes* samples cluster together, respectively, while Edo-dokoro (Hachi-1) is located in between the clusters of *D. tokoro* and *D. tenuipes* (**Fig. 4**). This result suggests a possibility that Edo-dokoro is a hybrid between *D. tokoro* and *D. tenuipes*.

**Fig. 5:**
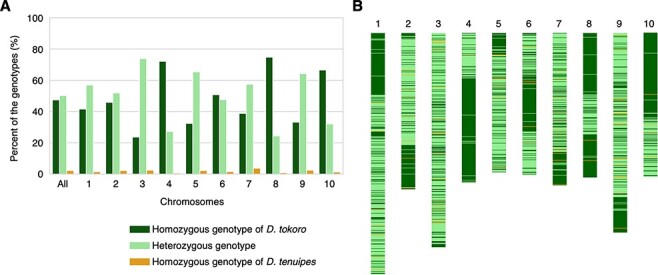
Genotype frequencies suggest that Edo-dokoro (Hachi-1) is derived from a hybrid between *D. tokoro* and *D. tenuipes*. (A) The genotype frequencies (homozygous for *D. tokoro*-specific nucleotides, heterozygous for *D. tokoro* and *D. tenuipes* nucleotides, and homozygous for *D. tenuipes*-specific nucleotides) show that heterozygous genotypes for *D. tokoro* and *D. tenuipes* nucleotides are abundant. In this analysis, we only focused on the nucleotides that are discriminatory between *D. tokoro* (three individuals) and *D. tenuipes* (three individuals). (B) Distribution of the genotypes shows that they are clustered like segments in chromosomes. The color codes correspond to that of **Fig. 5A**.

Next, we focused on a total of 915,083 biallelic SNPs that are discriminatory between *D. tokoro* and *D. tenuipes*, i.e. these are fixed to different nucleotides in the two species, and addressed their state in Edo-dokoro (Hachi-1). We found 460,070 sites (50.28%) that are heterozygous between the *D. tokoro*- and *D. tenuipes*-specific nucleotides, 434,253 sites (47.46%) that are homozygous for *D. tokoro*-specific nucleotides and 20,760 sites (2.27%) that are homozygous for *D. tenuipes*-specific nucleotides (**Fig. 5**; **Table 1**). Chromosome 3 containing a sex-determination locus in *D. tokoro* (Natsume et al. 2022) had a higher rate of heterozygosity (73.84%) than other chromosomes (**Fig. 5A**). This abundance of heterozygous SNPs in Edo-dokoro (Hachi-1) again suggests that Edo-dokoro is likely a hybrid between *D. tokoro* and *D. tenuipes*; however, it may not be a simple F1 between the two species, since the contribution from the *D. tokoro* genome is far larger than that from the *D. tenuipes* genome.

**Table 1:** Likelihood test shows that the BC1F1 model is the most likely for the origin of Edo-dokoro (Hachi-1). ‘A’ represents a nucleotide identical to that of *D. tokoro*, and ‘a’ represents a nucleotide identical to that of *D. tenuipes*. The observed frequency of a/a, 0.02, is set as an error rate. We only focused on the nucleotides which were discriminatory between *D. tokoro* and *D. tenuipes*. The numbers in the parentheses are the number of observed genotypes in the Edo-dokoro genome.

		Genotypes				
	Model	A/A	A/a	a/a	ln *L*(θ)	*P*-value
*Observation*		0.47 (434,253)	0.50 (460,070)	0.02 (20,760)		
*Expectation*	BC1F1	${{{\rm{1 - }}{p_{error}}{\ }} \over 2}$	${{{\rm{1 - }}{p_{error}}{\ }} \over 2}$	${p_{error}}$	−547.19	–
	BC2F1	${{3({\rm{1 - }}{p_{error}}{\rm{) }}} \over 4}$	${{({\rm{1 - }}{p_{error}}{\rm{) }}} \over 4}$	${p_{error}}$	−143,368.97	<0.0001

We also focused on the distribution of Edo-dokoro genotypes on each chromosome (**Fig. 5B**). This analysis showed that the homozygous SNPs for *D. tokoro*-specific nucleotides and the heterozygous SNPs between the *D. tokoro*- and *D. tenuipes*-specific nucleotides are clustered into two to four segments per chromosome. The small number of segments per chromosome suggests that Edo-dokoro has not experienced frequent hybridization after the first cross between *D. tokoro* and *D. tenuipes*. Considering the genotype frequencies in Edo-dokoro (**Fig. 5A**), it is possible that Edo-dokoro have experienced only single or double backcrosses with *D. tokoro* after the first cross between *D. tokoro* and *D. tenuipes*.

To address the possibility that Edo-dokoro was derived from the F1 hybrid backcrossed with *D. tokoro*, we calculated the likelihood of the observed SNP states for the two models, BC1F1 and BC2F1 (**Table 1**). The likelihood of BC1F1 (a single backcross to *D. tokoro* after crossing *D. tokoro* with *D. tenuipes*) is defined as $lnL\left( {{\theta _{BC1F1}}} \right)$, and the likelihood of BC2F1 (a double backcross to *D. tokoro* after crossing *D. tokoro* with *D. tenuipes*) is defined as $lnL\left( {{\theta _{BC2F1}}} \right)$.The log-likelihood value for the BC1F1 model was $lnL\left( {{\theta _{BC1F1}}} \right)$ = −547.19, whereas that for the BC2F1 model was $lnL\left( {{\theta _{BC2F1}}} \right) = - 143368.97$. The likelihood of the BC1F1 model was significantly (*P* < 0.0001) higher than that of the BC2F1 model, suggesting that Edo-dokoro is likely BC1F1 derived from a cross between the F1 and *D. tokoro*.

### Organellar DNA analysis shows the maternal origin of Edo-dokoro

**Fig. 6:**
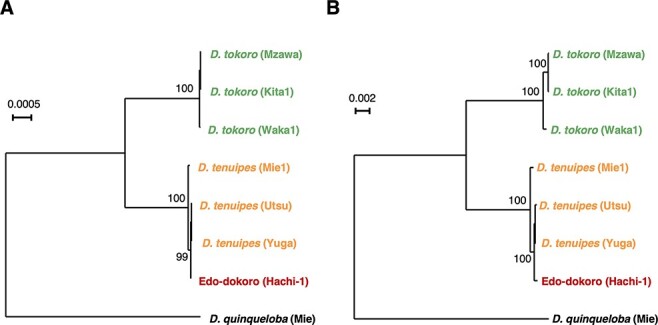
Organellar genome sequences of Edo-dokoro (Hachi-1) are grouped to those of *D. tenuipes* but not *D. tokoro*. (A) A phylogenetic tree of chloroplast sequences of *D. quinqueloba* (outgroup), *D. tokoro, D. tenuipes* and Edo-dokoro (Hachi-1). (B) A phylogenetic tree of mitochondrial sequences of *D. quinqueloba* (outgroup), *D. tokoro, D. tenuipes* and Edo-dokoro (Hachi-1). Both trees were reconstructed by IQ-TREE with 1,000 bootstraps.

**Fig. 7:**
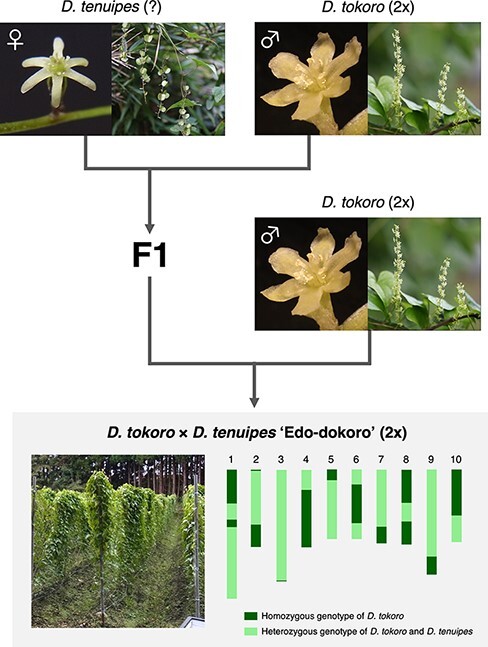
A hypothesized scheme of the origin of Edo-dokoro. Edo-dokoro is likely derived from a hybridization between a female *D. tenuipes* and a male *D. tokoro*, followed by a single backcrossing with a male *D. tokoro*. Bottom right: The ploidy of *D. tenuipes* crossed with diploid *D. tokoro* is still unknown, but flow cytometry shows that Edo-dokoro is diploid. The illustration of the genome map of Edo-dokoro, obtained by smoothing the graphical genotype shown in **Fig. 5B**.

From the whole-genome sequence data of a *D. tokoro* sample Kita1, we extracted the chloroplast (154 kb) and mitochondrial (575 kb) genome sequences. The whole-genome sequences of Edo-dokoro (Hachi-1), three *D. tokoro* individuals (Kita1, Mzawa and Waka1), three *D. tenuipes* individuals (Mie1, Utsu and Yuga) and an individual of *D. quinqueloba* were mapped to these reference genomes, and SNPs were called. Based on 829 polymorphic sites on the chloroplast genome and 1,193 polymorphic sites on the mitochondrial genome, we separately reconstructed DNA phylogenetic trees for the chloroplast and mitochondria (**Fig. 6**). Both chloroplast and mitochondrial genome trees clearly show that Edo-dokoro is grouped to *D. tenuipes*, but not to *D. tokoro*, suggesting that the female parent of Edo-dokoro is *D. tenuipes* (**Fig. 7**). Interestingly, the organellar sequences of Edo-dokoro clustered with those of *D. tenuipes* from the Eastern Japan (Yuga and Utsu; bootstrap value > 99%) but were distant from Central Japan (Mie1), suggesting that the F1 hybridization between *D. tokoro* and *D. tenuipes* may have happened in Eastern Japan.

## Discussion

This study revealed that a *Dioscorea* cultivar grown and traded in the Hachinohe region in Japan is most likely ‘Edo-dokoro’ as described in the botanical book of Makino (1940) and Satake et al. (1982). The name ‘Edo-dokoro’ appears in books published around 1700. It may be possible that the Hachi-1 individual we analyzed is one of the lineages of ‘Edo-dokoro’ vegetatively propagated over 300 years. ‘Edo-dokoro’ is called ‘Tokoro’ by the people in Hachinohe, and it is not acrid and is considered as a delicacy among the local people. The name of the cultivar indicates that Edo-dokoro originated from Tokyo, which was called ‘Edo’ during the Edo period (1603–1868). Horticulture was in full bloom during the Edo period, and people generated many varieties of *Camellia*, peony, *Chrysanthemum, Acer, Prunus, Iris, Nelumbo, Adonis, Primula, Lilium, Rohdea, Psilotum, Ardisia, Dendrobium* and *Ipomoea* (Nagase 2011). Therefore, it could be possible that Edo-dokoro has been generated by horticulturalists in the Edo period by crossing *D. tokoro* and *D. tenuipes*. The organellar genomes of Edo-dokoro are genetically closer to those of *D. tenuipes* from the Eastern Japan rather than from Central Japan (**Fig. 6**), which is in line with this hypothesis. In either case, considering the habitat of *D. tenuipes* (**Fig. 1**), it is likely that Edo-dokoro has spread from the place of origin and now is found only in the Aomori region. Then, the original name ‘Edo-dokoro’ has been forgotten, and the vernacular name ‘Tokoro’ was adapted to indicate this crop.

Our genome study using the nuclear genome as well as the organellar genomes revealed that Edo-dokoro was derived from a hybridization between female *D. tenuipes* and male *D. tokoro*, followed by a backcross with male *D. tokoro* (**Fig. 7**). Previous genetic studies have also revealed the domestication process and the origin of *Dioscorea* species (Bousalem et al. 2006, 2010, Scarcelli et al. 2006, 2017, 2019, Chaïr et al. 2010, 2016, Girma et al. 2014, Arnau et al. 2017, Siadjeu et al. 2018, Sharif et al. 2020, Sugihara et al. 2020, Bredeson et al. 2022), and some of them have reported the interspecific hybridizations in yams. For example, *D. rotundata* is most likely derived from a homoploid hybridization between *D. abyssinica* and *D. praehensilis* in Africa (Sugihara et al. 2020). Natural hybrid cultivars between *D. alata* and *Dioscorea nummularia* have been reported in Vanuatu (Chaïr et al. 2016). In Benin, traditional farmers’ practice called ‘ennoblement’, collecting tubers of wild yams from the bush and forest and planting them in their fields, likely contributes to the genetic diversity of cultivars through hybridization and introgression (Jarvis and Hodgkin 1999, Scarcelli et al. 2006, Chaïr et al. 2010). However, most findings are limited to the species in tropical regions. This study showed the hybrid origin of the cultivar Edo-dokoro in temperate regions.

Despite our findings in Edo-dokoro, no hybrids have been reported in the genus *Dioscorea* in Japan (Noda et al. 2022). By contrast, a recent taxonomic study has concluded that hybridization and reticulate evolution unlikely appeared in Japanese *Dioscorea* species, based on the relationships among species inferred from both plastid and nuclear regions (Noda et al. 2022). In this study, we showed the interspecific crossability between two *Dioscorea* species in Japan. Similar hybridization may have taken place in nature. Further investigations are needed to elucidate whether the hybridizations are limited to the process of domestication or if they also take place in wild species.

Although the domestication processes in several yams have been elucidated in the past decade, the key traits and genes involved in the domestication of yam were not identified. We have succeeded, in a cross between female Edo-dokoro and male *D. tokoro*, to obtain a large number of F1 progeny that are segregating for rhizome shape and acridness. We may be able to identify genomic regions controlling the edible traits of Edo-dokoro in future work. Also, we may be able to select a better cultivar with higher productivity and richer nutrition from the segregants for the ‘Renaissance’ of this neglected crop.

In summary, this study found that local farmers are propagating the vegetatively propagated crop Edo-dokoro over innumerable generations. Genomic study not only showed the hybrid origin of Edo-dokoro but also reconstructed the whole-genome map of Edo-dokoro (**Fig. 7**). Given that many of the valuable genetic resources maintained by small and local farmers are considered to be in danger of extinction, the survey of minor crops in local areas is of imminent importance globally.

## Materials and Methods

### Plant materials

One Edo-dokoro, three *D. tokoro* and three *D. tenuipes* were used in this research (**Supplementary Table S2**; **Fig. 1**). The rhizomes of Edo-dokoro were obtained from farmers in Tohoku-machi of Aomori. After growing these rhizomes in a greenhouse of IBRC for 7 years, a female individual of Edo-dokoro (sample ID: Hachi1) was selected for whole-genome sequencing. We used two whole-genome sequences of *D. tokoro* (sample ID: Kita1 and Waka1) published in the previous research (Natsume et al. 2022). Kita1 was sampled at Kitakami city of Iwate. Waka1 was sampled at Kushimoto city of Wakayama. In addition to these two sequences, we sequenced another male individual (sample ID: Mzawa) sampled at Mutsuzawa city of Chiba. We sequenced two male individuals (sample ID: Mie1 and Utsu) and one female individual (sample ID: Yuga) of *D. tenuipes*. Mie1 was sampled at Misugi city of Mie. Utsu was sampled at Hachioji city of Tokyo. Yuga was sampled at Yugawara city of Kanagawa. As an outgroup species, we sequenced *D. quinqueloba* (sample ID: Mie) sampled at Iinan-cho Matsusaka City of Mie (**Supplementary Table S2**).

### Flow-cytometry analysis

Flow-cytometry analysis was performed on Edo-dokoro (Hachi1) and *D. tokoro* (Kita1) with *P. hybrida* cv. Mitchell as an internal reference standard. Nuclei were isolated and stained with propidium iodide simultaneously and analyzed using a CyteFLEX System (Beckman Coulter, Brea, CA, USA) following the manufacturer’s protocol. We compared the genome sizes of Edo-dokoro (Hachi1) and *D. tokoro* (Kita1) using *P. hybrida* cv. Mitchell (~1.4 Gb) as an internal standard. The resulting data were analyzed by a python library FlowCytometryTools v0.5.1 (https://eyurtsev.github.io/FlowCytometryTools).

### Whole-genome resequencing

In addition to two whole-genome sequences of *D. tokoro* (Kita1 and Waka1) published in the previous research (Natsume et al. 2022), we resequenced six samples including one Edo-dokoro (Hachi1), one *D. tokoro* (Mzawa), three *D. tenuipes* (Mie1, Utsu and Yuga) and one *D. quinqueloba* (Mie) as an outgroup (**Supplementary Table S2**). Genomic DNA was extracted using NucleoSpin Plant II (MACHEREY-NAGEL GmbH & Co. KG, Düren, Germany). Genomic DNA of Hachi1 was extracted from fresh leaves, and the others were from leaves that were freeze dried with ELEYA FDL-2000 (Tokyo RIikakikai Co., Ltd., Tokyo, Japan) or sufficiently dried with silica gel. Then, DNA was quantified using Qubit (Invitrogen, Carlsbad, CA, USA). For Edo-dokoro (Hachi1), library construction and 150 bp paired-end sequencing were performed following the manufacturer’s protocol using the DNBSEQ platform (MGI Tech Co., Ltd, Shenzhen, China). For Mzawa, Yuga and Utsu, library constructions were performed using Collibri PCR-free ES DNA Library Prep Kits for Illumina Systems (Invitrogen, Carlsbad, CA, USA), and for the others, the TruSeq DNA PCR-Free Library Prep Kit (Illumina, San Diego, CA, USA) was used. These seven libraries were sequenced using the Illumina platform (Illumina, San Diego, CA, USA), for 150 bp paired-end reads.

### Quality control, alignment and SNP calling

We removed adapters using FaQCs v2.08 (Lo and Chain 2014). Then, we used PRINSEQ lite v0.20.4 (Schmieder and Edwards 2011) to remove low-quality bases at both ends using moving averages with window size = 5 and base quality average = 20. The large number of the short reads of Hachi1 and Kita1 are randomly resampled to reduce the number of short reads for computationally efficient SNP calling (**Supplementary Table S2**). The short reads of all eight samples were aligned to the reference genome of *D. tokoro* (*D. tokoro* Pseudo_Chromosome with unanchored contigs; 1,818 contigs; 444,733,147 bp) (Natsume et al. 2022) using bwa-mem2 v2.0pre2 (Vasimuddin et al. 2019) with default parameters (**Supplementary Table S2**). Then, we only retained properly paired reads using SAMtools v1.12 (Danecek et al. 2021) with the options ‘-f 0x2’. SNP calling was performed using BCFtools v1.9 (Danecek et al. 2021). At first, the ‘mpileup’ command in BCFtools was run with the following option set: ‘-B -q 10 -Q 13 -C 50 -I’. After that, the ‘call’ command in BCFtools was run with the option ‘-vm’. Finally, the ‘filter’ command in BCFtools was run with the option ‘i ‘INFO/MQ>=10’.

### Reconstruction of neighbor-net

We reconstructed a neighbor-net by SplitsTree5 (Huson and Bryant 2006). For this analysis, we used only biallelic SNPs with no missing genotype across all samples. The SNPs in the variant call format (VCF) were concatenated as a FASTA file. At that time, heterozygous SNPs were converted to International Union of Pure and Applied Chemistry (IUPAC) ambiguity codes. Finally, the FASTA file was input to SplitsTree5 v5.3.0 (Huson and Bryant 2006) to reconstruct a neighbor-net.

### Calculation of nucleotide diversity and *F*_ST_

We calculated the nucleotide diversity (${\hat \theta _\pi }$) in each population of *D. tokoro* and *D. tenuipes*, assuming that all individuals of *D. tenuipes* are diploid. We only used biallelic SNPs having no missing genotype in a population. We defined ${\hat \theta _\pi }$ as:

$${\hat \theta _\pi } = {1 \over {\bar L}}{n \over {n - 1}}{{\mathop \sum \nolimits_{i \lt j} {k_{ij}}} \over {n\left( {n - 1} \right)/2}}$$

where $\bar L$ is the number of average mapped sites in a population, $n$ is the number of chromosomes and ${k_{ij}}$ is the number of nucleotide differences between the $i$th and $j$th sequences. We also calculated Wright’s *F*_ST_ (Wright 1949) between the populations of *D. tokoro* and *D. tenuipes.* We only used biallelic SNPs having no missing genotype in both populations. The calculated *F*_ST_ were averaged in the genome.

### Test for hybridization based on genotype frequencies

Genotype frequencies of the generated VCFs were used to test for hybridization. The sequence of *D. quinqueloba* was not used in this analysis. Therefore, first, we removed the sequence of *D. quinqueloba* from the VCFs and retained only the sequences of seven individuals in the VCF: three individuals of *D. tokoro*, one individual of Edo-dokoro and three individuals of *D. tenuipes*. Second, only biallelic SNPs with no missing genotype in all seven sequences were retained. In addition, we retained only SNPs that were fixed in the opposite direction in the sequences of *D. tokoro* (three individuals) and *D. tenuipes* (three individuals). Finally, we counted the genotypes of Edo-dokoro at these positions. The ‘All’ in **Fig. 5A** includes markers on the unanchored contigs that are not part of chromosomes 1–10.

### Test for backcrossing

Confidence interval based on likelihood ratio was used to validate the backcrossing between Edo-dokoro and *D. tokoro*. In this analysis, we used the markers of Edo-dokoro used in ‘Test for the hybridization based on genotype frequencies’. The likelihood of BC1F1 (a single backcross to *D. tokoro* after crossing *D. tokoro* with *D. tenuipes*) is defined as:

$$\begin{aligned}L\left( {{\theta _{BC1F1}}} \right) & = {{\left( {{N_{AA}} + {N_{Aa}} + {N_{aa}}} \right)!} \over {{N_{AA}}! \times {N_{Aa}}! \times {N_{aa}}!}} \times {\left( {{{1 - {p_{error}}} \over 2}} \right)^{{N_{AA}}}} \\ & \quad \times {\left( {{{1 - {p_{error}}} \over 2}} \right)^{{N_{Aa}}}} \times {p_{error}}^{{N_{aa}}}\end{aligned}$$

and the likelihood of BC2F1 (a double backcross to *D. tokoro* after crossing *D. tokoro* with *D. tenuipes*) is defined as:

$$\begin{aligned}L\left( {{\theta _{BC2F1}}} \right) & = {{\left( {{N_{AA}} + {N_{Aa}} + {N_{aa}}} \right)!} \over {{N_{AA}}! \times {N_{Aa}}! \times {N_{aa}}!}} \times {\left\{ {{{3\left( {1 - {p_{error}}} \right)} \over 4}} \right\}^{{N_{AA}}}} \\ & \quad \times {\left( {{{1 - {p_{error}}} \over 4}} \right)^{{N_{Aa}}}} \times {p_{error}}^{{N_{aa}}}\end{aligned}$$

where *N_AA_, N_Aa_* and *N_aa_* represent the number of each observed genotype of Edo-dokoro (*N_AA_*: 434,253 markers, *N_Aa_*: 460,070 markers and *N_aa_*: 20,760 markers, respectively). *N_AA_* is the number of observed homozygous genotypes of *D. tokoro* (A/A) in the Edo-dokoro genome. *N_Aa_* is the number of observed heterozygous genotypes of *D. tokoro* and *D. tenuipes* (A/a) in the Edo-dokoro genome. *N_aa_* is the number of the observed homozygous genotypes of *D. tenuipes* (a/a) in the Edo-dokoro genome. The observed frequency of a/a, 0.02, is set as an error rate *p_error_* derived from the violation of the assumption that the used markers are fixed in the opposite directions in both the *D. tokoro* and *D. tenuipes* populations. By comparing the two likelihoods, we found that the single backcross model ($lnL\left( {{\theta _{BC1F1}}} \right) = - 547.19$) explains the observation significantly better than the double backcross model ($lnL\left( {{\theta _{BC2F1}}} \right) = - 143368.97$) does (*P* < 0.000000000001).

### Reconstruction of phylogenetic tree of chloroplast and mitochondria

To obtain the chloroplast and mitochondrial sequences of *D. tokoro* (Kita1), we performed a de novo assembly using Illumina short reads and Nanopore long reads sequenced by Natsume et al. (2022). First, base-calling of the Nanopore long reads was performed for FAST5 files using Guppy 5.0.16 (Oxford Nanopore Technologies, Oxford, UK). Next, the Nanopore long reads, converted to FASTQ format, were assembled using NECAT v0.0.1 (Chen et al. 2021). To further improve the accuracy of the assembly, Racon v1.4.20 (Vaser et al. 2017) was used twice for error correction, and Medaka v1.4.1 (https://github.com/nanoporetech/medaka) was subsequently used to correct mis-assembly. Following this, two rounds of consensus correction were performed using bwa-mem v0.7.17 (Li 2013) and HyPo v1.0.3 (Kundu et al. 2019) with Illumina short reads, resulting in a 417 Mb de novo assembly comprising 431 contigs. We then aligned Nanopore long reads to these contigs using minimap2 v2.17 (Li 2018) and calculated the depth of the reads aligned to each contig using CoverM v0.6.1 (https://github.com/wwood/CoverM). We selected nine contigs with greater than 200 depth as mitochondria and two with greater than 9,000 depth as chloroplast because those with high read depths were more likely to be organelle genomes. The long reads aligned to these contigs were collected as the reads derived from the chloroplast and mitochondrial genome, respectively, and each was assembled using NECAT again. As a result, a single sequence of 154 kb (CP) was obtained from the chloroplast reads. For mitochondrial reads, three contigs were generated, but we retained the longest sequence of 463 kb as the mitochondrial sequence (MT) based on the results of the blastn search at National Center for Biotechnology Information (NCBI).

We also performed Racon and Medaka for the raw contigs before bridging in the above NECAT process of whole-genome de novo assembly and subsequently obtained 417 contigs with haplotigs removed using purge-haplotigs v1.1.1 (Roach et al. 2018). After adding CP and MT to these 417 contigs and performing NECAT bridge command, the 419 contigs were combined into 203. Following this, we performed two rounds of consensus correction using bwa-mem and HyPo with Illumina short reads. Finally, we obtained a 398 Mb de novo assembly of *D. tokoro* (Kita1), including a 154 kb chloroplast sequence and a 575 kb mitochondrial sequence.

Using this 398 Mb FASTA file as a reference, we generated a VCF file the same way as the ‘SNP calling’ section using the sequence reads obtained in the ‘Whole-genome resequencing’ section above. After generating the VCF, we only retained the markers having no missing and heterozygous genotype across all eight samples on the chloroplast and mitochondrial region. Based on the pattern of these markers, we created concatenated multi FASTA files for the chloroplast and mitochondria. Using these FASTA files, the phylogenetic trees of the chloroplast and mitochondria were reconstructed by IQ-TREE v1.6.11 (Nguyen et al. 2015) with 1,000 ultrafast bootstrap analysis (Hoang et al. 2018). The models for each tree were selected by ModelFinder (Kalyaanamoorthy et al. 2017) implemented in IQ-TREE v1.6.11 (Nguyen et al. 2015). As a result, the ‘K3P+ASC’ and ‘TVMe+ASC’ models were selected for the chloroplast and mitochondria, respectively. Finally, the phylogenetic trees were drawn by SeaView v5.0.4 (Gouy et al. 2010).

## Supplementary Material

pcac109_Supp

## Data Availability

All sequencing read data generated for this work have been deposited in the DNA Databank of Japan database under BioProject PRJDB13110 and PRJDB12945; see **Supplementary Table S2** for individual sample accession numbers. *Dioscorea tokoro*’s chloroplast and mitochondrial fastas are available at the following URL: https://genome-e.ibrc.or.jp/resource/dioscorea-tokoro/. The datasets and scripts used in this study are available in the Github repository (https://github.com/ncod3/Edo-dokoro_scripts/).
