# Supplementary material for: Genome Analysis Revives a Forgotten Hybrid Crop Edo-dokoro in the Genus Dioscorea
Source: Plant Cell Physiol. 2022 Jul 25;63(11):1667–78. doi: 10.1093/pcp/pcac109 (PMC9680860; doi:10.1093/pcp/pcac109)
Supplement: pcac109_Supp [file pcac109_supp.zip › pcp-2022-e-00073-File009.pdf]

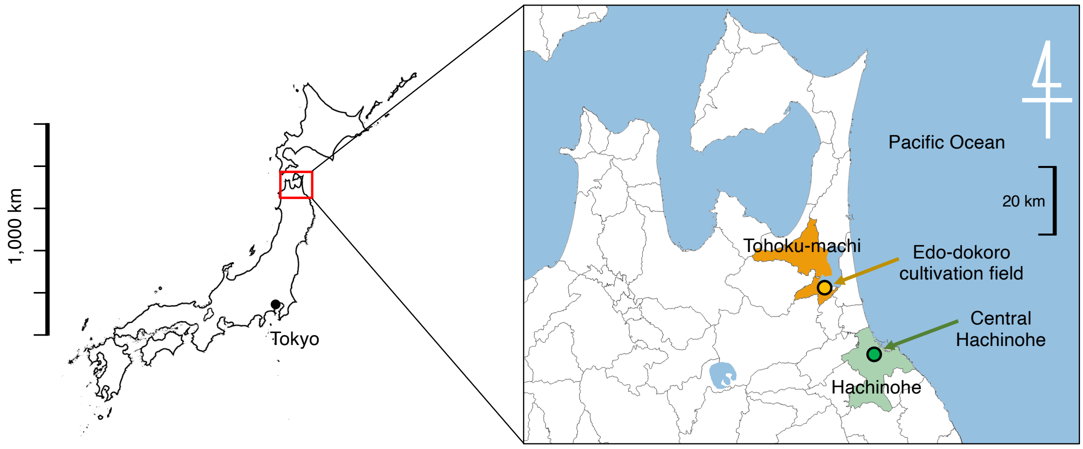

Fig. S1 An enlarged map of Hachinohe region of Northern Honshu Island where Hachi-1 sample is cultivated. The orange area is Tohoku-machi, and the orange circle indicates the Edo-dokoro cultivation field. The green area is Hachinohe city, and the green circle indicates Central Hachinohe, where the supermarket sells Edo-dokoro. Tohoku-machi is a small town with a population of about 16,000, and Hachinohe City is the second-largest city in Aomori Prefecture, with about 220,000. The distance between the two points is about 30km. The base map and data were obtained the Ministry of Land, Infrastructure, Transport and Tourism (https://nlftp.mlit.go.jp/ksj/gml/datalist/KsjTmplt-N03-v3_0.html#prefecture00), All Japan (N03-20210101_GML.zip). The map was created with Quantum Geographic Information System (QGIS) software version 3.16.0 (QGIS Development Team, 2022).

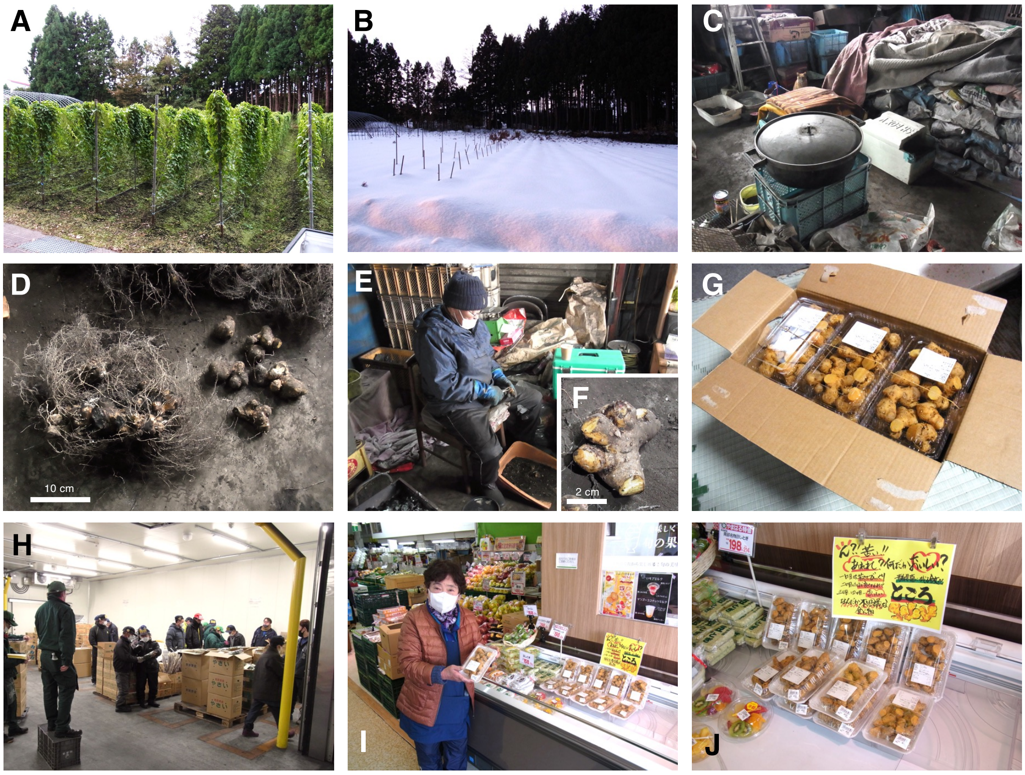

Fig. S2 A Japanese minor crop Edo-dokoro (Hachi-1) is cultivated and consumed in Aomori Prefecture in Japan. (A) A field of Edo-dokoro in October just before harvest. This field is 20m x 50m in size. Four farmers cultivate Edo-dokoro in Tohoku-machi. (B) Tohoku-machi is snowed in during the winter from December to March. Farming work during this period is limited and only indoor works are possible. (C) The rhizomes of Edo-dokoro are put into bags with the soil and stacked in the warehouse (right). The big pot is to boil Edo-dokoro rhizomes. (D) Rhizome covered with the fibrous roots (left) is harvested about six to eight months after the seed rhizome was planted (right). (E) During the snowy season from January to March, the work of cutting the fibrous roots of the harvested rhizome continues. The fibrous roots are carefully removed with scissors so that no hard fibrous roots remain when eaten. (F) Rhizomes are cut into bite-sized pieces. (G) After boiling in a large pot for about two to four hours, the rhizomes are placed in plastic cases. (H) Edo-dokoro rhizomes are auctioned off at a wholesale market. (I) The owner of a major supermarket in Hachinohe City purchased Edo-dokoro and immediately displayed it. This supermarket has sold Edo-dokoro for many years. (J) Edo-dokoro lined up on the display in the supermarket. Japanese advertisement says 'Hmm? That's bitter! What? Is it good? The first day, you'll be surprised at how bitter it is; the second day, you'll get used to it; the third and fourth day, you'll be surprised at how ...... yummy! It's a very strange food.'

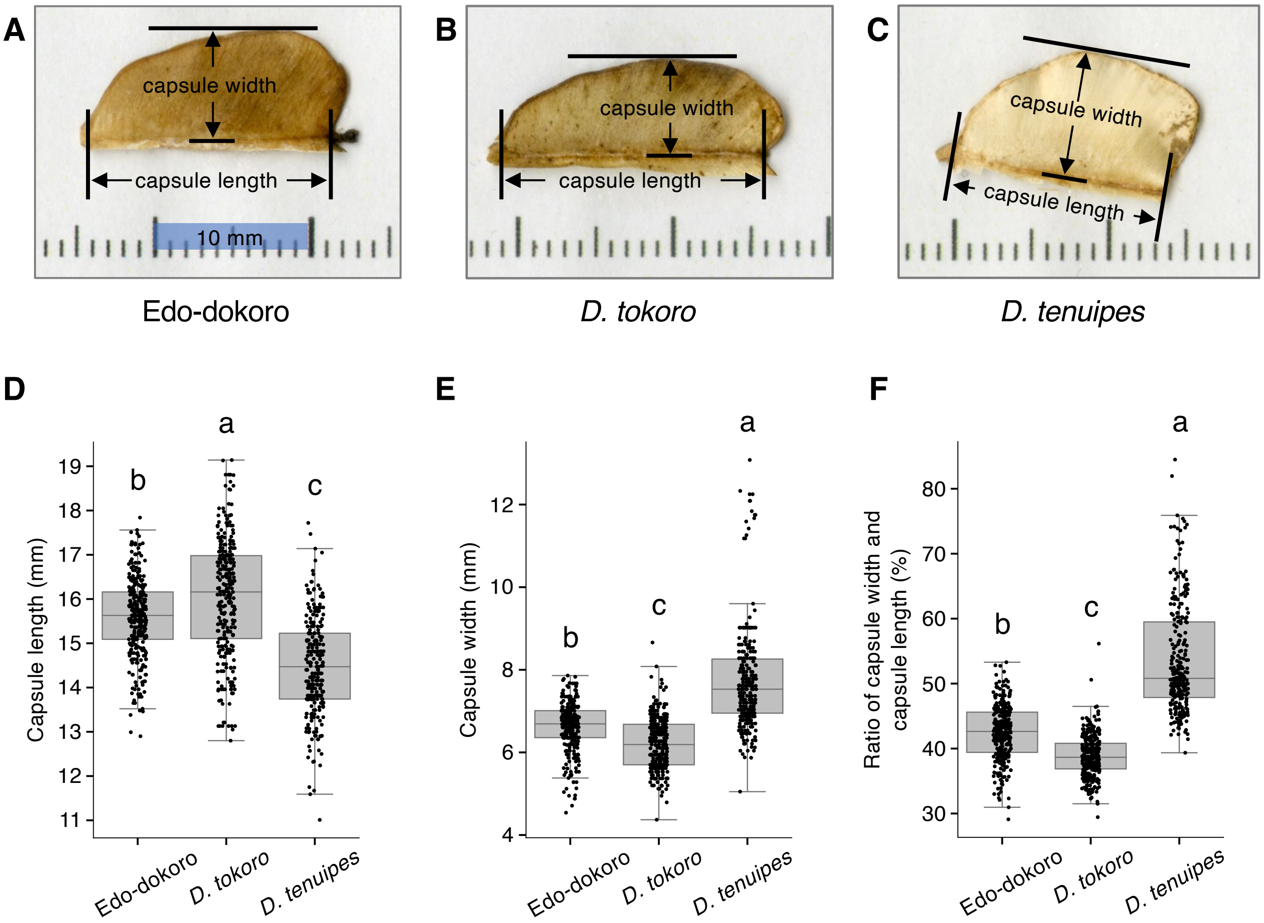

Fig. S3 Comparison of sizes of capsular fruits of Edo-dokoro, *D. tokoro* and *D. tenuipes*. Three capsular characters were compared among the three species. Capsules of (A) Edo-dokoro, (B) *D. tokoro*. (C) *D. tenuipes*. (D) Distributions of capsule length. (E) Distributions of capsule width. (F) Distributions of the ratio of capsule width and capsule length (%). Mean values were compared using Steel-Dwass test. Error bar indicates 1.5 × the interquartile range. The boxplots with the same letter indicate no significant difference at $\alpha = 0.05$.

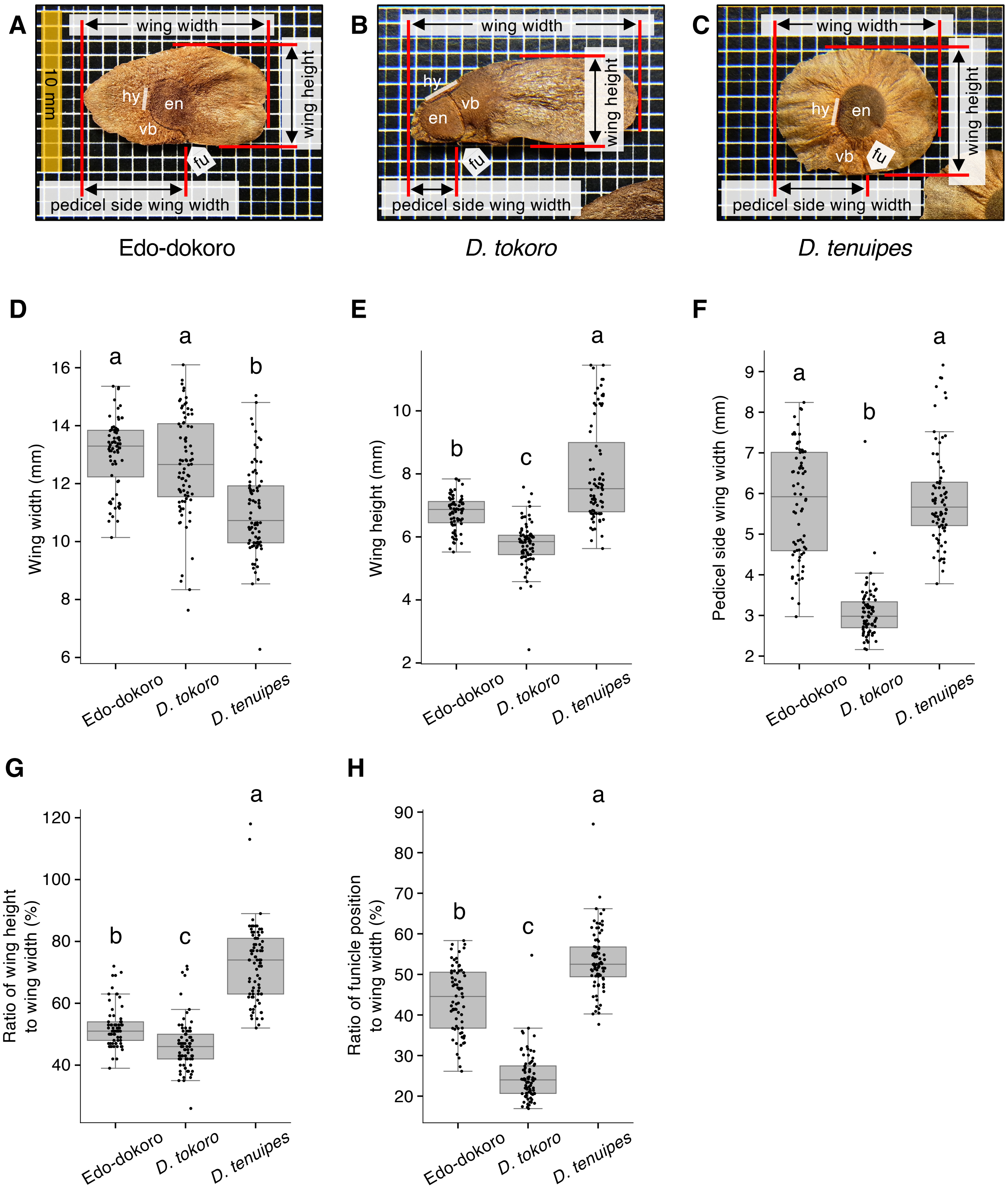

Fig. S4 Comparison of seed size in Edo-dokoro, *D. tokoro* and *D. tenuipes*. Five seed characters were compared among the three species. Seeds of (A) Edo-dokoro, (B) *D. tokoro* and (C) *D. tenuipes*. fu: funicle, vb: vascular bundle, hy: hypostase, en: endsperm. (D) Distributions of wing width. (E) Distributions of wing height. (F) Distributions of pedicel side wing width. (G) Distributions of the ratio of wing height to width. (H) Distributions of the ratio of funicle position to wing width. Mean values were compared using Steel-Dwass test. Error bar indicates 1.5× the interquartile range. The boxplots with the same letter indicate no significant difference at $\alpha = 0.05$.

Table S1 List of samples used for measurement of sizes of fruits and seeds.

Species	Sampling place	No. of capsules of fruit [c]	Total no. capsules in the species	Seed no.	Total no. seeds in the species	Sampling date
Edo-dokoro	IBRC Field [a]	300	300	20	72	16 Nov 2020
	Crossed in IBRC (1) [b]			20		2 Dec 2014
	Crossed in IBRC (2) [b]			12		2 Dec 2014
	Crossed in IBRC (3) [b]			20		2 Dec 2014
D. tokoro	Hanamaki, Iwate	100	300	20	80	27 Nov 2013
	Matsushima, Miyagi	100		20		2 Nov 2013
	Takizawa, Iwate	100		20		10 Nov 2013
	Koka, Shiga			20		15 Nov 2020
D. tenuipes	Hachioji, Tokyo	15	274	20	80	5 Dec 2021
	Machida, Tokyo	59		20		5 Nov 2021
	Yugawara, Kanagawa	100		20		6 Nov 2021
	Iwakura, Kyoto	100		20		10 Jan 2017
	Total		874		232	

[a] Fruits resulted by natural mating between Edo-dokoro and wild males of *D. tokoro* in the IBRC field.
[b] Fruits resulted by an artificial cross between Edo-dokoro and a male of *D. tokoro* in the IBRC greenhouse.
[c] Capsules of mature fruits were used.

Table S2 Summary of the sequences of four *Dioscorea* species and the status of the alignment to the reference genome of *D. tokoro* (Kita1).

Species	Sample ID	Sex	Sampling information			FASTQ size (G-bp)		Status of alignment [d]				Accession ID
			Date	Latitude	Longitude	Before filtering	After filtering	Coverage (%) [e]		Average depth [f]		
								Depth ≥ 1	Depth ≥ 5	Depth ≥ 1	Depth ≥ 5	
**Edo-dokoro**	Hachi-1	Female	Mar. 2014	40.72	141.29	53.7	11.2 [b] (53.6)	94.3	88.4	13.6	20.5	DRR351751
D. tokoro	Kita1	Male	Jun. 2011	39.28	140.89	37.8	12.5 [b] (37.4)	99.9	99.1	23.8	24.0	DRR347075
	Waka1	Female	Unknown [a]	33.54	135.86	12.6	12.3	93.7	90.5	23.5	24.2	DRR351752
	Mzawa	Male	Aug. 2020	35.34	140.31	12.2	11.6	95.1	90.8	19.0	19.7	DRR351753
D. tenuipes	Yuga	Male	Nov. 2021	35.13	139.06	18.5	17.8	71.7	58.0	26.2	31.9	DRR351755
	Utsu	Female	Nov. 2021	35.63	139.33	17.5	16.9	73.4	57.8	25.0	31.2	DRR351756
	Mie1	Male	Jul. 2018	34.51	136.37	11.1	10.8	67.0	49.3	13.6	17.6	DRR351754
D. quinqueloba	Mie	Unknown	Jul. 2018	34 [c]	136 [c]	13.2	12.8	45.9	25.7	11.6	19.0	DRR351757

[a] Waka1 is an individual used in the previous research (Terauchi et al. 1997).
[b] The short reads of Hachi-1 and Kita1 are randomly resampled for computationally efficient SNP calling.
[c] *D. quinqueloba* (Mie) was sampled at Iinan-cho Matsuzaka City of Mie, but there is no other information.
[d] The genome size used here is 443,496,847 bp as a result of excluding the number of ambiguous "N" (1,236,300 bps) from the size of the genome (Pseudo_Chromosome with unanchored contigs, 444,733,147 bps) have used in the analysis.
[e] "Coverage" is defined as how widely short reads cover the genome (i.e., mapped sites / genome size excluded the number of "N" that appear as ambiguous nucleotide). For mapped sites, two thresholds are set (depth >= 1 and >= 5).
[f] "Average depth" is defined as the average number of reads on one mapped site (i.e., Total number of nucleotides of aligned reads / mapped sites). For mapped sites, two thresholds are set (depth >= 1 and >= 5).

Table S3 Summary of the number of SNPs and sample sets investigated in each analysis.

Analysis	No. SNPs	Species*	Values
$\hat{\theta}_\pi$ (*D. tokoro*)	3,113,287	to	0.00392
$\hat{\theta}_\pi$ (*D. tenuipes*)	1,496,981	te	0.00376
F_{ST} (*D. tokoro* vs. *D. tenuipes*)	2,926,393	to/te	0.534
Fig. 4	1,054,866	to/te/qu/edo	-
Fig. 5	915,083	to/te/edo	-
Table 1	915,083	to/te/edo	-
Fig. 6A (Chloroplast)	829	to/te/qu/edo	-
Fig. 6B (Mitochondria)	1,193	to/te/qu/edo	-

* to: *D. tokoro*, te: *D. tenuipes*, qu: *D. quinqueloba*, and edo: Edo-dokoro

Supplementary methods:

Measurement of morphological characters and statistical analysis

Morphological measurement were performed on capsules of fruits (Fig. S3) and seeds (Fig. S4). 874 capsules of fruits and 232 seeds were collected in total from Edo-dokoro, *D. tokoro* and *D. tenuipes* (Table S1). In capsules of fruits, we compared three characters: placenta length, capsule height, and ratio of capsule height and placenta length (%). In seeds we compared five characters: wing width, wing height, pedicel side wing width, ratio of the wing height to wing width, and ratio of the funicle position to the wing width.

Statistical analysis was performed using python version 3.10.4. To check that each dataset were normally distributed, Shapiro-Wilk test was used. To confirm homogeneity of variance, Levene test was used. All datasets were not normal distribution or homogeneity of variance. Means were compared using Kruskal-Wallis test followed by Steel-Dwass test (non-parametric analysis). All statistical significances were set at $p < 0.05$.

The function used and the included python library are as follows: Shapiro-Wilk test (shapiro function from stats library of scipy package), Levene test (levene function from stats library of scipy package), Kruskal-Wallis test (kruskal function from stats library of scipy package), Steel-Dwass test (posthoc_dscf function from scikit_posthocs package).
